# Early-stage bilayer tissue-engineered skin substitute formed by adult skin progenitor cells produces an improved skin structure in vivo

**DOI:** 10.1186/s13287-020-01924-z

**Published:** 2020-09-18

**Authors:** Qun Zhang, Jie Wen, Chang Liu, Chuan Ma, Fuxiang Bai, Xue Leng, Zhihong Chen, Zhiwei Xie, Jun Mi, Xunwei Wu

**Affiliations:** 1 Department of Tissue Engineering and Regeneration, School and Hospital of Stomatology, Cheeloo College of Medicine, Shandong University & Shandong Key Laboratory of Oral Tissue Regeneration & Shandong Engineering Laboratory for Dental Materials and Oral Tissue Regeneration, No.44-1 Wenhua Road West, Jinan, Shandong China; 2 Shandong Provincial Key Laboratory of Animal Cells and Developmental Biology, School of Life Science, Shandong University, Qingdao, China; 3 Qilu Children’s Hospital of Shandong University, Jinan, China; 4 Department of Stomatology, Shengli Oilfield Center Hospital, Dongying, Shandong China

**Keywords:** Tissue-engineered skin, Skin progenitor cell, Skin regeneration, Hair follicle regeneration, p63

## Abstract

**Background:**

In recent years, significant progress has been made in developing highly complex tissue-engineered skin substitutes (TESSs) for wound healing. However, the lack of skin appendages, such as hair follicles and sweat glands, and the time required, are two major limitations that hinder its broad application in the clinic. Therefore, it is necessary to develop a competent TESS in a short time to meet the needs for clinical applications.

**Methods:**

Adult scalp dermal progenitor cells and epidermal stem cells together with type I collagen as a scaffold material were used to reconstitute bilayer TESSs in vitro. TESSs at 4 different culture times (5, 9, 14, and 21 days) were collected and then grafted onto full-thickness wounds created in the dorsal skin of athymic nude/nude mice. The skin specimens formed from grafted TESSs were collected 4 and 8 weeks later and then evaluated for their structure, cell organization, differentiation status, vascularization, and formation of appendages by histological analysis, immunohistochemistry, and immunofluorescent staining.

**Results:**

Early-stage bilayer TESSs after transplantation had a better efficiency of grafting. A normal structure of stratified epidermis containing multiple differentiated layers of keratinocytes was formed in all grafts from both early-stage and late-stage TESSs, but higher levels of the proliferation marker Ki-67 and the epidermal progenitor marker p63 were found in the epidermis formed from early-stage TESSs. Interestingly, the transplantation of early-stage TESSs produced a thicker dermis that contained more vimentin- and CD31-positive cells, and importantly, hair follicle formation was only observed in the skin grafted from early-stage TESSs. Finally, early-stage TESSs expressed high levels of p63 but had low expression levels of genes involved in the activation of the apoptotic pathway compared to the late-stage TESSs in vitro.

**Conclusions:**

Early-stage bilayer TESSs reconstituted from skin progenitor cells contained more competent cells with less activation of the apoptotic pathway and produced a better skin structure, including hair follicles associated with sebaceous glands, after transplantation, which should potentially provide better wound healing when applied in the clinic in the future.

## Background

Skin injuries from accidents, diseases, chronic wounds, acute trauma, burns, etc. can compromise the skin barrier and potentially lead to permanent disability or death of the injured person depending on the severity of the wound. According to the latest statistics, 450,000 burn injuries require medical attention annually in the USA, and 40,000 of those patients require hospitalization and the cost is significant [1]. Skin grafting is one of the most promising approaches to heal extensive wounds [2], and without grafts, full-thickness skin wounds with diameters larger than 4 cm are difficult to heal [3]. However, autografts are not always available, particularly for burn patients. In only very limited numbers of cases, patients have suitable autografts for transplantation. For allografts, the demand for skin tissues seriously exceeds the supply, and moreover, foreign tissues have the additional problem of potential immune rejection. Since tissue engineering was formally introduced in the late 1980s, tissue-engineered skin substitutes (TESSs) have become an attractive solution to treat acute and chronic cutaneous wounds [4, 5]. The common method used to create TESSs involves seeding a biodegradable scaffold with cells including epidermal keratinocytes, dermal fibroblasts, and/or stem cells. In recent decades, human TESSs have evolved from simple epidermal substitutes to complex full-thickness skins with a bilayer containing both epidermal and dermal layers that are also commercially available for clinical applications [6–9]. However, there is still much room for improvement given the many practical and therapeutic limitations of TESSs at this time [6].

On one hand, the complete regeneration of perfect skin with full functions (protection, regulation, and sensation) is still challenging and has become a major aim in wound healing [10]. Although current TESSs have been improved by the implementation of various appendages, including a capillary network [11], sensory innervation [12], adipose tissue [13], and pigment production [14], it remains impossible to reliably reconstitute full skin functions, most notably functional hair follicles and sweat glands [15]. The failure of cultured cells to regenerate hair follicles has been ascribed to the loss of trichogenicity during expansion in culture [16–18]. Therefore, it is crucial for effective regenerative medicine to develop an optimal system to maintain cell regeneration potential in vitro. The availability of adult stem cells and induced pluripotent stem cells (iPSCs) from patients provides opportunities to eventually generate those structures without the risk of immune rejection [19, 20]. On the other hand, since extensive cell culture procedures are involved in producing the different cell types used, the reconstruction of full-thickness TESSs is very time-consuming. Cells required for epidermal and dermal components of bilayer TESSs usually require 2 to 4 weeks of culture to obtain sufficient numbers of cells to produce TESSs, which then need another 3 or more weeks of culture before they are ready for grafting, which is a major constraint for their regular use as an autologous product for treating wounds in the clinic. Therefore, shortening the culture time required both for cell expansion and for reconstitution of TESSs would have a significant benefit for the clinical application of autologous TESSs.

Recently, we established a simple and quick method to efficiently isolate human skin epidermal and dermal stem cells [21] and to shorten the initial culture time required for the expansion of those skin progenitor cells [22]. Importantly, these human skin cells after expansion in our system still maintain their potential to generate full-thickness skin with hair follicles [23]. Here, we used our method to prepare skin progenitor cells to reconstruct bilayer TESSs and explored whether early-stage TESSs with a short culture time were able to form skin structures similar to late-stage TESSs in vivo after transplantation (Additional file 1: Figure S1).

## Materials and methods

### Preparation of skin progenitor cells

Skin progenitor cell preparations followed the procedures described in our previous publications [21, 22, 24]. Human epidermal stem cells and human dermal progenitor cells were derived from adult scalp tissues (males, age 20–30 years old). The adult scalp tissues were collected from discarded hospital specimens following methods approved by the Medical Ethical Committee of the School of Stomatology, Shandong University (No. 2015120401; date: 05 December 2015), without any personal identity information. The epidermal stem cells were cultured in K-SFM (Cat. 10725-018, Thermo Fisher Scientific, Waltham, MA, USA) plus 5 μM Y-27632 (Cat. Y0503, Sigma-Aldrich, St. Louis, MO, USA), and the medium was changed every other day. The dermal progenitor cells were cultured in DMEM (Cat. 12430054, Thermo Fisher Scientific)/F12 (Cat. 21127022, Thermo Fisher Scientific) (3:1) containing 5% fetal bovine serum (FBS, Cat. 16140071, Thermo Fisher Scientific), 0.1% penicillin/streptomycin (Cat. 10378016, Thermo Fisher Scientific), 20 ng/ml EGF (Cat. PHG0311, Thermo Fisher Scientific), 40 μg/ml Fungizone (Cat. 15290026, Thermo Fisher Scientific), and 2% B27 supplement (Cat. 17504-044, Thermo Fisher Scientific), and the growth medium was changed every 5 days. Epidermal and dermal cells used were previously frozen cells at passage 1, and after thawing, the cells were cultured for two more passages (passage 3) to obtain sufficient numbers of cells for all experiments. These dermal and epidermal progenitor cells have been proven to be multipotent after culture expansion [22, 25].

### Construction of bilayer TESSs

Bilayer TESSs are composed of a stratified epithelial layer containing differentiated epidermal cells on the top of a dermal layer containing differentiated dermal fibroblasts mixed with collagen matrix and was constructed according to a previously described protocol [26]. A schematic of the production of TESSs is shown in Additional file 1: Figure S1a-f.

1. First, an acellular collagen layer is established that acts as an attachment substrate for the cellular layer (Additional file 1: Figure S1a) and prevents the cellular collagen from contracting from the insert membrane and detaching from it. Preparing the acellular collagen mixture on ice prevents premature gelation: mix 0.57 ml 10× MEM (Cat. 61100087, Thermo Fisher Scientific), 3.4 ml 2.5 mg/ml bovine type I collagen (Cat. 804592, Sigma-Aldrich), 0.83 ml FBS, 55 μl 200 mM l-glutamine (Cat. G7513, Sigma-Aldrich), and 2.0 ml DMEM, then adjust the pH to 7.2 with saturated NaHCO_3_ (Cat. S5761, Sigma-Aldrich). Use chilled pipets and avoid creating air bubbles when mixing. Add 1 ml of that mixture to each well of 6-well tissue culture plate transwells, ensuring that the gel coats the entire transwell bottom. The plates are then cultured at 37 °C, 5% CO_2_ in an incubator, and the mixture will gel in 30 min.
2. Second, a collagen matrix with dermal progenitor cells is constructed on top of the acellular collagen layer (Additional file 1: Figure S1b). Dermal progenitor cells are resuspended to a final concentration of 2 × 10^5^ cells/ml in DMEM. Mix 1.65 ml 10× MEM, 6.5 ml 2.5 mg/ml bovine type I collagen, 1.8 ml FBS, 165 μl 200 mM l-glutamine, and 2.0 ml DMEM, then adjust the pH to 7.2 with saturated NaHCO_3_, add 6.75 ml 2 × 10^5^ fibroblasts/ml, and mix the ingredients together thoroughly. Add 3 ml of that mixture into each insert on top of the gelled acellular collagen matrix and incubate the plates at 37 °C, 5% CO_2_ in an incubator. When the cellular matrix is completely gelled after 60 min, add 10 ml fibroblast culture medium to each well around the insert and 2 ml directly in the insert. Incubate the matrix and allow it to contract until day 4 (Additional file 1: Figure S1c).
3. Third, establish the epidermal cell layer (Additional file 1: Figure S1d). After contraction is complete and the matrix is stabilized at day 4, adult epidermal stem cells are added to the surface of the matrix. A total of 2 × 10^6^ keratinocytes in 100 μl DMEM per insert should be seeded directly onto the contracted collagen gels, then incubated for 60 min at 37 °C (without medium) to allow the keratinocytes to fully adhere. Epidermal cells are allowed to attach to the substrate to generate a confluent cellular monolayer that will initiate tissue stratification.
4. Fourth, culture different stages of TESSs. Add 12 ml epidermalization medium to each insert according to a previously described protocol [26], 10 ml to the bottom and 2 ml on top. Change the medium every 2 days until day 11 (Additional file 1: Figure S1e). The tissues are then raised to the air-liquid interface at day 12 to enable complete stratification and changed to the air-liquid epidermalization medium by adding 7 ml medium to the bottom of the well and ensuring that the insert just contacts the medium every other day until day 21 (Additional file 1: Figure S1f).

TESSs were collected at four different time points during the culture for in vitro and in vivo analyses, summarized in Additional file 1: Figure S1: (1) TESS-5d—the TESS was collected at day 5 (1 day after adding epidermal stem cells, Additional file 1: Figure S1d); (2) TESS-9d—the TESS was collected at day 9 (including 4 days of epidermalization medium culture, Additional file 1: Figure S1e); (3) TESS-14d—the TESS was collected at day 14 (including 6 days of epidermalization medium culture and 3 days of air-liquid interface culture, Additional file 1: Figure S1f); and (4) TESS-21d—the TESS was collected at day 21 (including 6 days of epidermalization medium culture and 10 days of air-liquid interface culture, Additional file 1: Figure S1f). The detailed culture information for each TESS is summarized in Additional file 1: Table S1.

### Grafting of TESSs in vivo

The grafting protocol for TESSs and a schematic diagram are shown in Additional file 1: Figure S2, modified from a previously published study [27–29]. Briefly, before grafting, a silicon membrane is placed over the insert and it is flipped over with the full-thickness model. Eight-week-old female athymic nude/nude mice (Cat. 403, Charles River, Beijing, China) were prepared for surgery and draped with betadine solution under anesthesia; 6 mice were used for each grafting group. An area around 2 cm^2^, which is similar to the size of the TESS, of dorsal skin intended to be grafted was excised in full thickness leaving the musculature beneath undamaged (Additional file 1: Figure S2a). Bleeding is controlled with gentle pressure, and the silicon membrane with the full-thickness TESS is flipped over onto the wound. The membrane is sutured to the host skin (Additional file 1: Figure S2b), and sterile dressings are applied to provide constant pressure against the graft to the wound bed (Additional file 1: Figure S2c). The mice are monitored every 2 days, and dressings are removed 8 days later. Mice were sacrificed to collect the grafts at 4 and 8 weeks for analysis.

### Hematoxylin and eosin, immunohistochemistry, and immunofluorescence staining

The grafted tissues were collected at the desired times and were fixed with 4% paraformaldehyde (PFA), then were embedded in paraffin blocks. Six-micrometer sections of paraffin-embedded tissues were made for H&E staining, IHC, and IF analyses following standard protocols [30–32]. The following staining kits were used according to the manufacturers’ supplied protocols: an Alcian blue-Periodic Acid Schiff (PAS) stain kit (Cat. ab245876, Abcam, Cambridge, MA, USA) that was used to stain the basement membrane and a Masson’s trichrome stain kit (Cat. G1340, Solarbio, Beijing, China) that was used to stain collagen fibers. The following primary antibodies were used: monoclonal mouse anti-human pan-cytokeratin (pan-ck, keratin 14/15/16/19, Cat. 550951, BD Biosciences, San Jose, CA, USA), polyclonal rabbit anti-Ki-67 (Cat. ab15580, Abcam), monoclonal mouse anti-human vimentin (Cat. 3390, Cell Signaling Technology, Danvers, MA, USA), polyclonal rabbit anti-keratin 10 (Cat. ab111447, Abcam), polyclonal rabbit anti-p63 antibody (Cat. ab53039, Abcam), rabbit anti-CD31 antibody (Cat. ab134168, Abcam), rabbit anti-β-catenin antibody (Cat. ab16051, Abcam), and rabbit anti-loricrin antibody (Cat. ab85679, Abcam). The following secondary antibodies were used: Alexa fluor-488 donkey anti-mouse IgG (Cat. ab150105, Abcam), Alexa fluor-594 donkey anti-mouse IgG (Cat. ab150108, Abcam), Alexa fluor-594 donkey anti-rabbit IgG (Cat. ab150076, Abcam), and goat anti-rabbit IgG H&L (Cat. ab205718, Abcam). Dilutions of 1:400 were used for all secondary antibodies. The stained slides were mounted with a mounting medium containing DAPI to stain the nuclei (Cat. ab104139, Abcam).

### Western blot analysis

Western blot analysis was done following standard protocols [33]. Briefly, TESSs were washed three times with ice-cold phosphate-buffered saline (PBS, Cat. 10010049, Thermo Fisher Scientific) and then lysed with radioimmunoprecipitation assay (RIPA, Cat. 89900, Thermo Fisher Scientific) buffer containing 1% phenylmethylsulfonyl fluoride (PMSF, Cat. 36978, Thermo Fisher Scientific) and 1% phosphatase inhibitor cocktails. Protein concentrations were determined using a bicinchoninic acid (BCA) protein quantitation kit (Cat. PC0020, Solarbio) following the manufacturer’s protocol. Twenty micrograms of each protein sample was loaded on SDS-PAGE gels then electro-transferred to polyvinylidene fluoride (PVDF) membranes (Cat. 88518, Thermo Fisher Scientific). The membranes were incubated with the specific primary antibodies overnight at 4 °C after incubation with blocking buffer (Cat. 37580, Thermo Fisher Scientific) for 1 h. The following day, the membranes were washed with TBST (Cat. T1081, Solarbio) and incubated with secondary antibodies for 4 h at room temperature. Detection was performed with chemiluminescence reagents (Cat. SW2050, Solarbio), and immunoreactive bands were quantified using ImageJ analysis. The following primary and secondary antibodies were used: rabbit anti-cleaved Caspase-3 antibody (Cat. 9664, Cell Signaling Technology), rabbit anti-Pro-Caspase-3 (Total Caspase-3) antibody (Cat. ab32499, Abcam), rabbit anti-cytokeratin 1 antibody (Cat. ab93652, Abcam), rabbit anti-cytokeratin 5 antibody (Cat. ab52635, Abcam), rabbit anti-p63 antibody (Cat. ab124762, Abcam), rabbit anti-GAPDH antibody (Cat. ab181602, Abcam), and HRP anti-rabbit (Cat. 7074, Cell Signaling Technology).

### qRT-PCR analysis

Total RNA was extracted from cells using a QIAGEN RNeasy Plus Mini Kit (QIAGEN, Germantown, MD, USA) according to the manufacturer’s instructions. The RNAs were dissolved in nuclease-free water, and their concentrations were measured using a Nanodrop spectrophotometer. qRT-PCR was carried out in 12.5 μl reaction volumes using a KAPA SYBR FAST One-Step Universal Kit (KAPA Biosystems, Woburn, MA, USA) with an ABI 7500 Fast System programmed as follows: 42 °C for 5 min, 95 °C for 1 min, and 40 cycles of PCR at 95 °C for 15 s, and 60 °C for 30 s. Data were acquired and analyzed with t 7500 Fast System SDS software. The human ribosomal gene 36B4 was used as a housekeeping gene for the internal control. The primers for each assessed gene were listed in Additional file 1: Table S2.

### Statistical analysis

For all quantification data, Student’s *t* test analysis was used when comparing an experimental group with a control group, and one-way or two-way ANOVA with correction for multiple pairwise comparisons was used when comparing more than two groups with one or two independent variables, respectively [34, 35]. All experiments were repeated with at least three technical replicates in each experiment, and typical representative experiments are shown in all cases. Error bars reported indicate standard errors of the means (SEMs), and *p* values < 0.05 are considered significant and are indicated in the figures with asterisks. Comparisons not marked were not significantly different.

## Results

### Better efficiency of grafting after transplantation of early-stage TESS

**Fig. 1:**
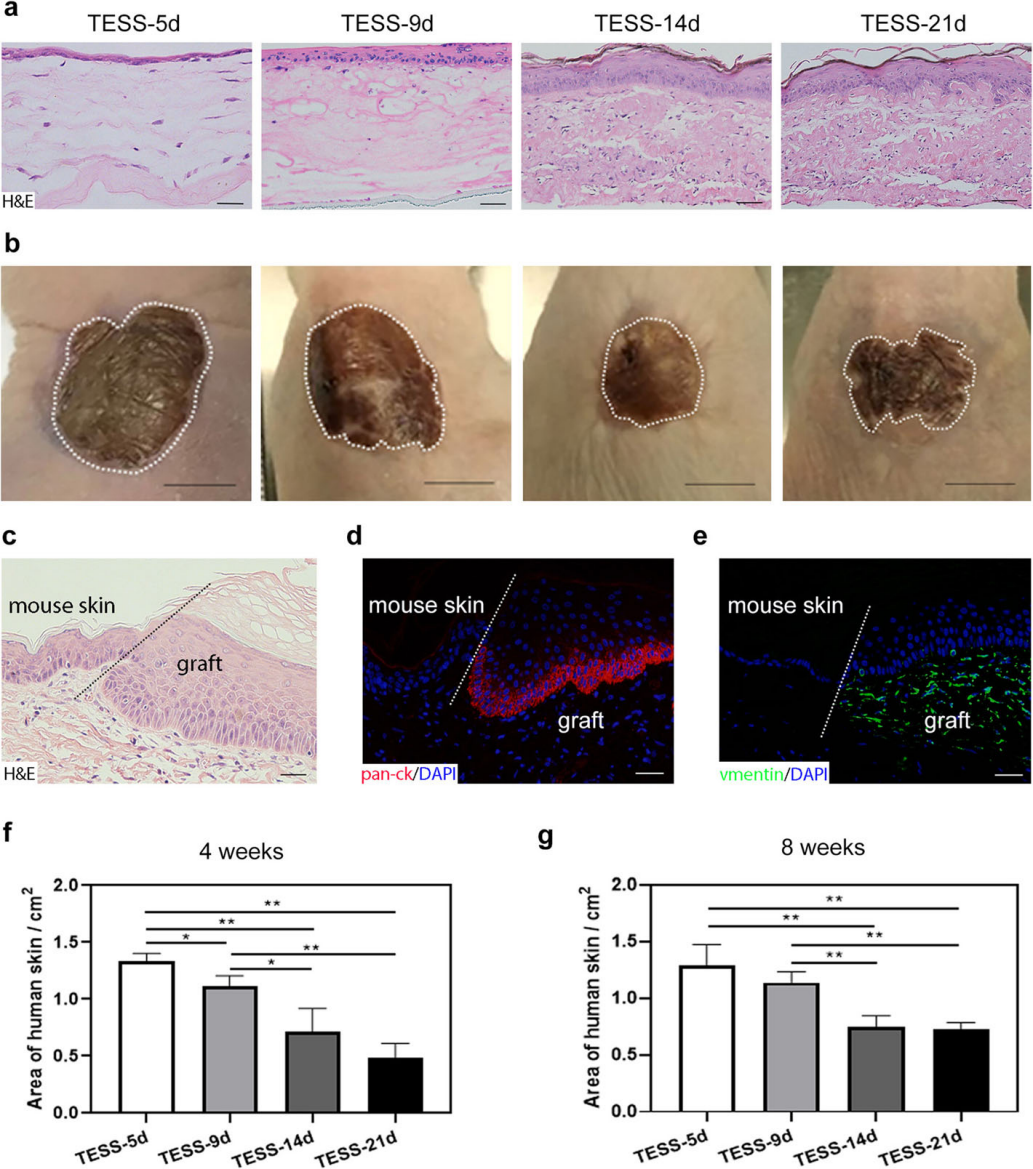
TESSs from different time points produce pigmented skin after grafting. **a** Representative H&E-stained images of TESSs at different time points (bars = 50 μm). **b** Representative images of the skin in graft areas at 4 weeks after transplantation of different TESSs (bars = 5 mm). The white dashed line indicates the border of the host mouse skin and the pigmented human graft skin area. **c** Representative image of H&E staining of the pigmented area from **b**. The black dashed line indicates the boundary between the human skin graft area and the host mouse skin. **d**, **e** IF staining of human pan-ck (red, **d**) and human vimentin (green, **e**) in the pigmented area from **b**; DAPI stains the nuclei (blue). The white dashed line indicates the boundary between the human skin graft and the host mouse skin (bars = 50 μm). **f**, **g** The average size of pigmented skin areas at 4 and 8 weeks after transplantation. *p < 0.05, **p < 0.01 when two groups were compared as indicated; none of the other comparisons was significantly different (3 mice for each group, *n* = 3)

To test whether different stages of TESSs affect the efficiency of grafting in vivo after transplantation, reconstituted TESSs from skin progenitor cells were collected at four time points: from early-stage (day 5 (TESS-5d) and day 9 (TESS-9d)) and from late-stage (day 14 (TESS-14d) and day 21 (TESS-21d)). Before the transplantation, the histology of those TESSs was assessed by H&E staining (Fig. 1a). H&E staining showed that TESS-5d only formed a thin monolayer of the epidermis, and with increased culture time, the epidermis became thicker and contained multiple layers of differentiated keratinocytes. The all-over thickness of the dermis was comparable in all 4 groups, but the dermis became denser with more matrix deposition in late-stage TESSs compared to TESS-5d.

TESSs with the same size (around 2 cm^2^, formed in 6-well plates) from each group were grafted on the dorsal skin of female athymic nude/nude mice. Four weeks after the transplantation, the successful surviving grafts were clearly visualized by pigmented areas (dashed lines, Fig. 1b) since the host skin of athymic nude/nude mice does not have functional melanocytes necessary to produce pigmentation. To further confirm that the pigmented area was derived from the grafted TESSs, the areas were collected and histological analysis was performed (with H&E, Fig. 1c) along with IF analysis using specific antibodies of human pan-ck for epidermal cells (Fig. 1d) and vimentin for dermal cells (Fig. 1e). H&E staining clearly showed the boundary between the thin mouse skin and the thick human skin (indicated by the black dashed line, Fig. 1c). That was confirmed by IF staining, which showed that the skin from the grafted area was positive for antibodies to human pan-ck and to human vimentin and did not stain the host mouse skin (indicated by the white dashed lines, Fig. 1d, e). To test the efficiency of grafting taken from the transplantation of different stages of TESSs, 6 pieces of TESSs for each time point were grafted to 6 mice individually. The results showed that all grafts (6 of 6, 100%) from TESS-5d and TESS-9d, and 5 of 6 grafts (83%) from TESS-14d and TESS-21d produced pigmented areas, showing no statistically significant difference between early-stage and late-stage TESSs. Furthermore, the size of the pigmented areas (indicated by the white dashed lines, Fig. 1b) was measured at 4 and 8 weeks after transplantation and revealed that early-stage TESSs produced larger pigmented areas after transplantation compared to late-stage TESSs (Fig. 1f, g). This result suggests that the transplantation of early-stage TESSs potentially gives a better efficiency of grafting.

### Normal differentiated epidermis is formed from the transplantation of different stages of TESSs

**Fig. 2:**
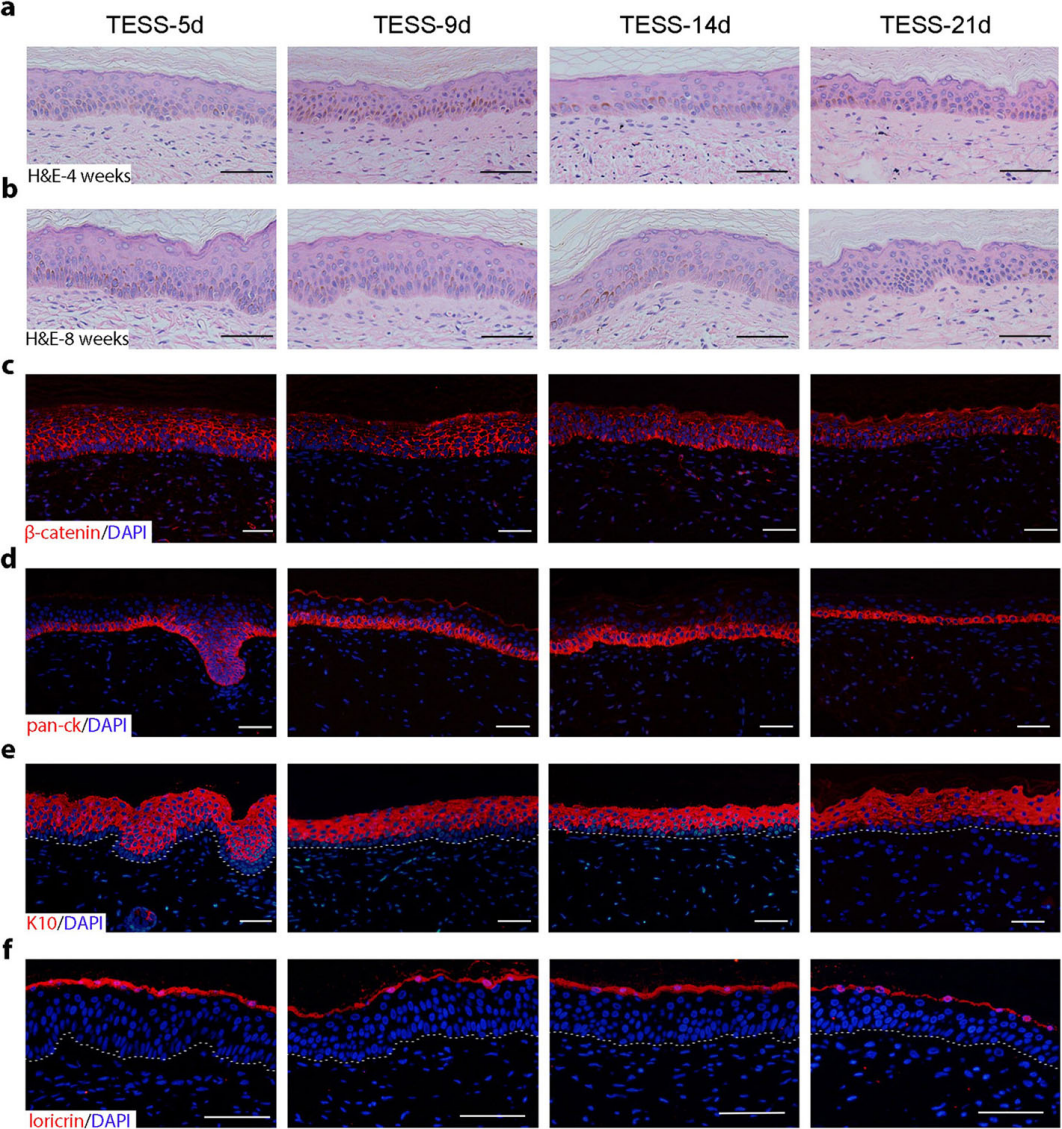
A normal structure of the epidermis is formed from transplantation of different stages of TESSs at 4 and 8 weeks after grafting. **a**, **b** Representative images of H&E staining of the epidermis formed at 4 (**a**) and 8 (**b**) weeks after grafting. **c**–**f** Representative images of IF staining of β-catenin (red, **c**), human pan-ck (red, **d**), K10 (red, **e**), and loricrin (red, **f**) at 4 weeks; DAPI stains the nuclei (blue); bars = 50 μm

To further characterize the structure of grafted skins formed from different stages of TESSs, we evaluated the histological structure of the epidermis formed at 4 and 8 weeks after transplantation. H&E staining showed that the formed epidermis (stratified epithelia) of all four groups contained multiple layers of keratinocytes, which were composed of two distinct layers according to their morphology: a polarized basal cell layer and a suprabasal layer containing spinous, granular, and cornified layers (Fig. 2a, b). Histological analysis showed no significant differences in epidermal architecture between 4 and 8 weeks for all TESSs, indicating that a normal and stratified epidermal structure was already formed at 4 weeks after grafting. Next, we examined the cell-cell organization in the formed epidermis using IF staining of β-catenin, a component of adherens junctions. Keratinocytes were well connected with each other in the epidermis, and there was no significant difference in β-catenin staining patterns among the 4 groups at 4 weeks (Fig. 2c).

In order to further confirm those histological results, the differentiation status of the formed epidermis at 4 weeks after transplantation was assessed by IF staining of different epidermal differentiation markers. First, pan-ck, an antibody that specifically binds to the human basal cell markers cytokeratins 14, 15, 16, and 19, was positive in the basal layer of the formed epidermis in all 4 groups (Fig. 2d) and also confirmed that the epidermal cells are of human origin. The suprabasal cell marker keratin 10 (K10) was localized in the suprabasal layer of the epidermis and was not present in the basal cell layer (Fig. 2e). Loricrin, a component of the stratum corneum, was localized at the top of the epidermis (Fig. 2f). These results show that the epidermis in the graft area had a normal architecture structure with a normal differentiation status. Taken together, these results suggested that early-stage TESSs with a thin layer of the epidermis (TESS-5d, Fig. 1a) produced a normally differentiated and stratified epidermis within 4 weeks after transplantation, and the overall thickness of the epidermis was equal to, or in some cases, even slightly thicker, than that formed from the transplantation of late-stage TESSs.

### Increased Ki-67 and p63 expression in the epidermis formed from early-stage TESSs

**Fig. 3:**
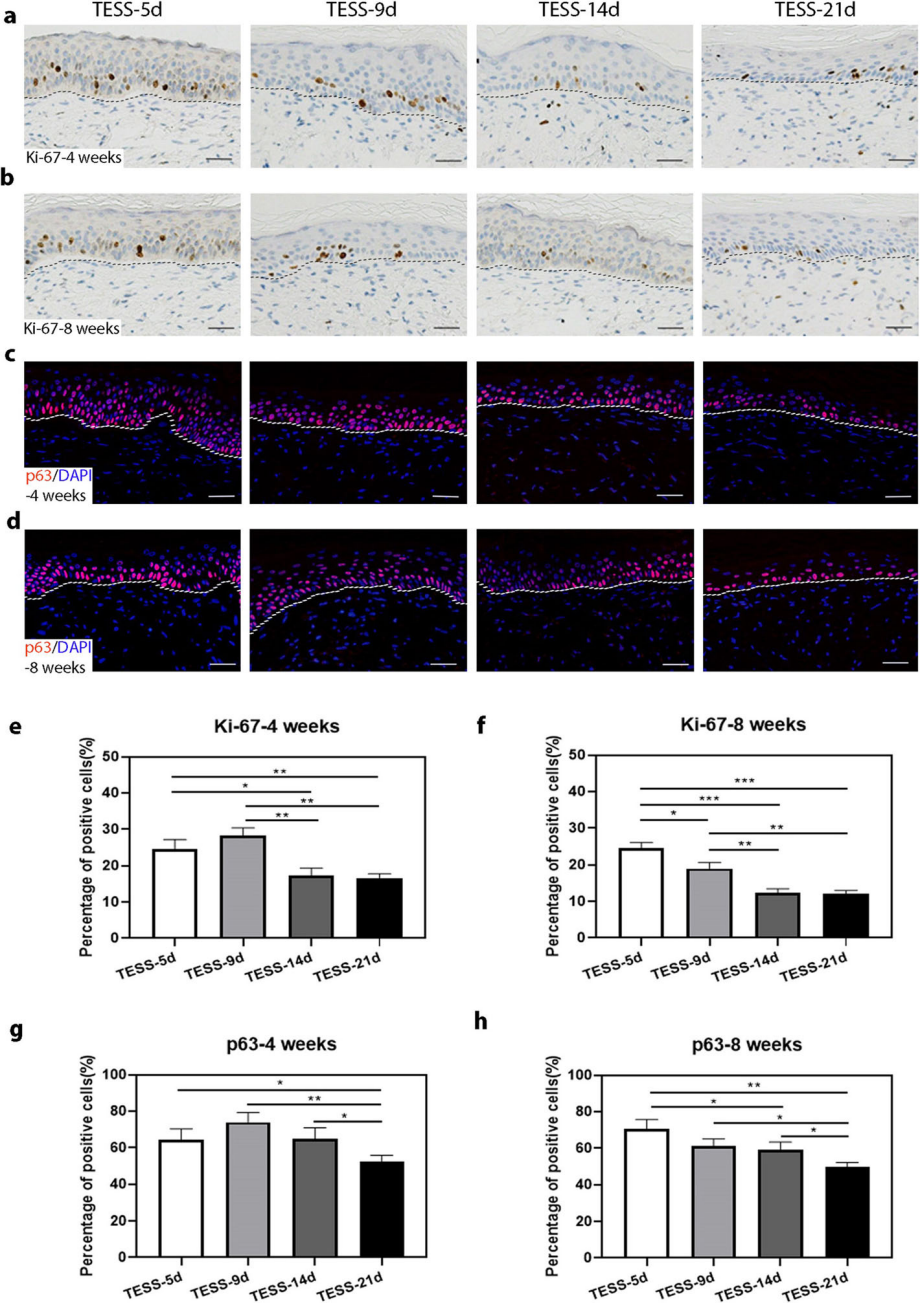
The epidermis from early-stage TESSs contains more Ki-67- and p63-positive cells. **a**, **b** Representative images of Ki-67 IHC staining of the epidermis formed 4 (**a**) and 8 (**b**) weeks after grafting different stages of TESSs. **c**, **d** Representative images of IF staining (red) of p63 in the epidermis formed 4 (**c**) and 8 (**d**) weeks after grafting different stages of TESSs; DAPI stains the nuclei (blue). **e**, **f** Quantification of the percentage of Ki-67-positive cells in the basal cell layer by counting the number of Ki-67-positive cells in a total of 200 basal cells with DAPI staining from **a** for 4 and **b** for 8 weeks after transplantation. **g**, **h** Quantification of the percentage of p63-positive cells in the epidermis formed by counting the number of p63-positive cells in a total of 200 epidermal cells with DAPI staining from **c** for 4 and **d** for 8 weeks after transplantation. *p < 0.05, **p < 0.01, ***p < 0.005 when two groups were compared as indicated; none of the other comparisons was significantly different (3 mice for each group, *n* = 3); bars = 50 μm

Early-stage TESS containing a monolayer epidermis developed into mature stratified epithelia similar to that formed from later-stage TESSs at 4 weeks after transplantation in vivo. We speculated that the epidermal cells might proliferate more rapidly in the epidermis formed from TESS-5d after transplantation. IHC staining of the proliferation marker Ki-67 for grafts at 4 and 8 weeks after transplantation (Fig. 3a, b) revealed that proliferating cells were mainly located in the basal layer of the epidermis in all groups as expected. Then, we performed IF staining of p63, which plays an essential role in the initial formation of the epidermis during development and also for mature keratinocytes to regenerate a stratified epithelium, and we observed much stronger staining of p63 in the basal cell layer among all groups (Fig. 3c, d). Quantification of the percentage of stained cells showed there were more Ki-67-positive proliferating cells in the epidermis formed from transplants of early-stage TESSs (TESS-5d or TESS-9d) compared to late-stage TESSs (TESS-14d or TESS-21d) (Fig. 3e, f). For quantification of p63-positive cells, overall, the number of p63-positive cells in the epidermis of early-stage TESSs (TESS-5d or TESS-9d) was higher than that of late-stage TESSs, especially higher than TESS-21d, although there was no significant difference between TESS-9d and TESS-14d, and there was no statistically significant difference between TESS-5d and TESS-9d as well (Fig. 3g, h). Taken together, these data suggested that epidermal cells from early-stage TESSs have more potential to proliferate and differentiate after transplantation.

### Thicker dermis appears in the skin formed from transplants of early-stage TESSs

**Fig. 4:**
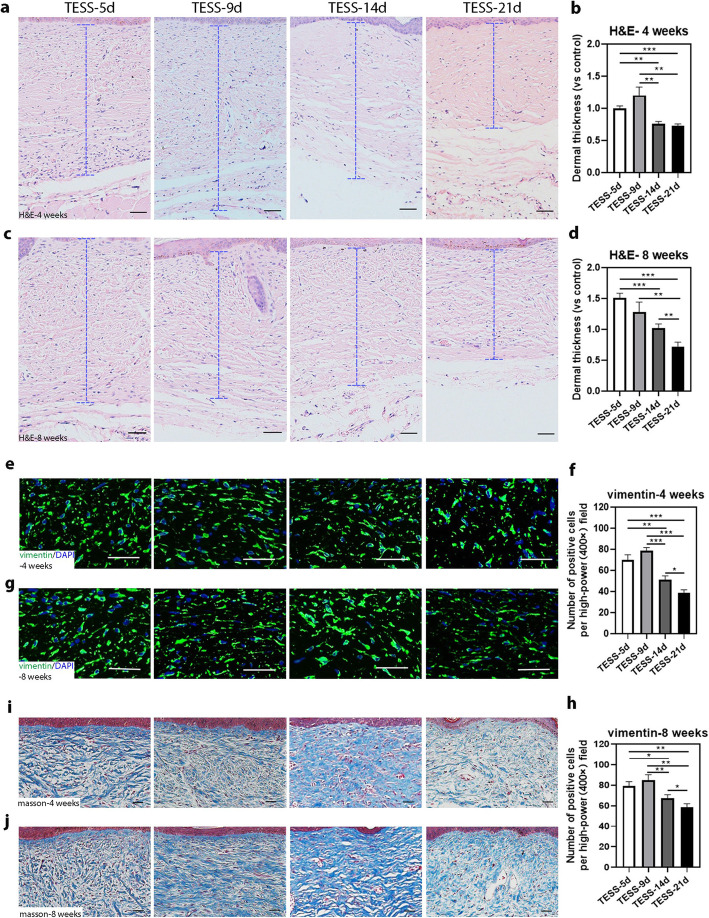
Thicker dermis appears in the skin formed from transplantation of early-stage TESSs. **a**–**d** Representative H&E-stained images of the dermis formed from the transplantation of different stages of TESSs at 4 (**a**) and 8 weeks (**c**) after transplantation. Blue dashed bars indicate the thickness of the dermis measured, and the averages of dermis thickness (*n* = 3) at 4 and 8 weeks are shown in **b** and **d**, respectively. **e**, **g** Representative images of IF staining of vimentin (green) in the dermis with DAPI staining (blue). **f**, **h** Corresponding quantification of vimentin-positive cells per high-power field (× 400) in a light microscope from **e** for 4 and **g** for 8 weeks after transplantation, respectively. **i**, **j** Representative images of Masson’s trichome staining of the dermis formed for collagen fibers (blue) from the transplantation of different stages of TESSs at 4 (**i**) and at 8 weeks (**j**) after transplantation. *p < 0.05, **p < 0.01, ***p < 0.005 when two groups were compared as indicated; none of the other comparisons was significantly different (3 mice for each group, *n* = 3); bars = 50 μm

Next, we characterized the dermis formed from transplants of different stages of TESSs. H&E staining showed that the overall structure of the dermis was not significantly different among all groups at 4 and 8 weeks after grafting (Fig. 4a–d). The thickness of the dermis was measured as indicated in Fig. 4a, c, and the average thickness is shown in Fig. 4b, d. Interestingly, these data showed that the dermis formed from early-stage TESSs was slightly thicker than the dermis formed from late-stage TESSs, and especially, TESS-21d produced the thinnest dermis compared to the other groups. Next, we examined the density of dermal cells in the formed dermis. H&E staining showed that there were more cells in the dermis formed from early-stage TESSs (Fig. 4a, b). To confirm that observation, we performed IF staining using an antibody to human vimentin, a mesenchymal cell marker, and confirmed that the dermal cells were of human origin (Fig. 4e, g). Quantification of vimentin-positive cells in the formed dermis revealed that there were more vimentin-positive cells in the dermis from early-stage TESSs than in late-stage TESSs (Fig. 4f, h). To further evaluate the distribution of dermal fibroblasts, Masson’s trichrome staining was carried out to analyze the organization of collagen fibers (blue stain) in the dermis. The distribution and structure of collagen fibers and bundles (stained blue) were comparable in TESSs of all groups, and the collagen fibers were organized and aligned in bundles in all groups, but collagen fibers of late-stage TESSs were slightly denser (Fig. 4i, j). Taken together, these results suggest that the structure of the dermis formed from the transplantation of early-stage TESSs is comparable to the dermis formed from late-stage TESSs, but early-stage TESSs produced a thicker dermis with more dermal cells after transplantation.

### An intact basement membrane is formed with more CD31-positive cells in the dermis of early-stage TESSs

**Fig. 5:**
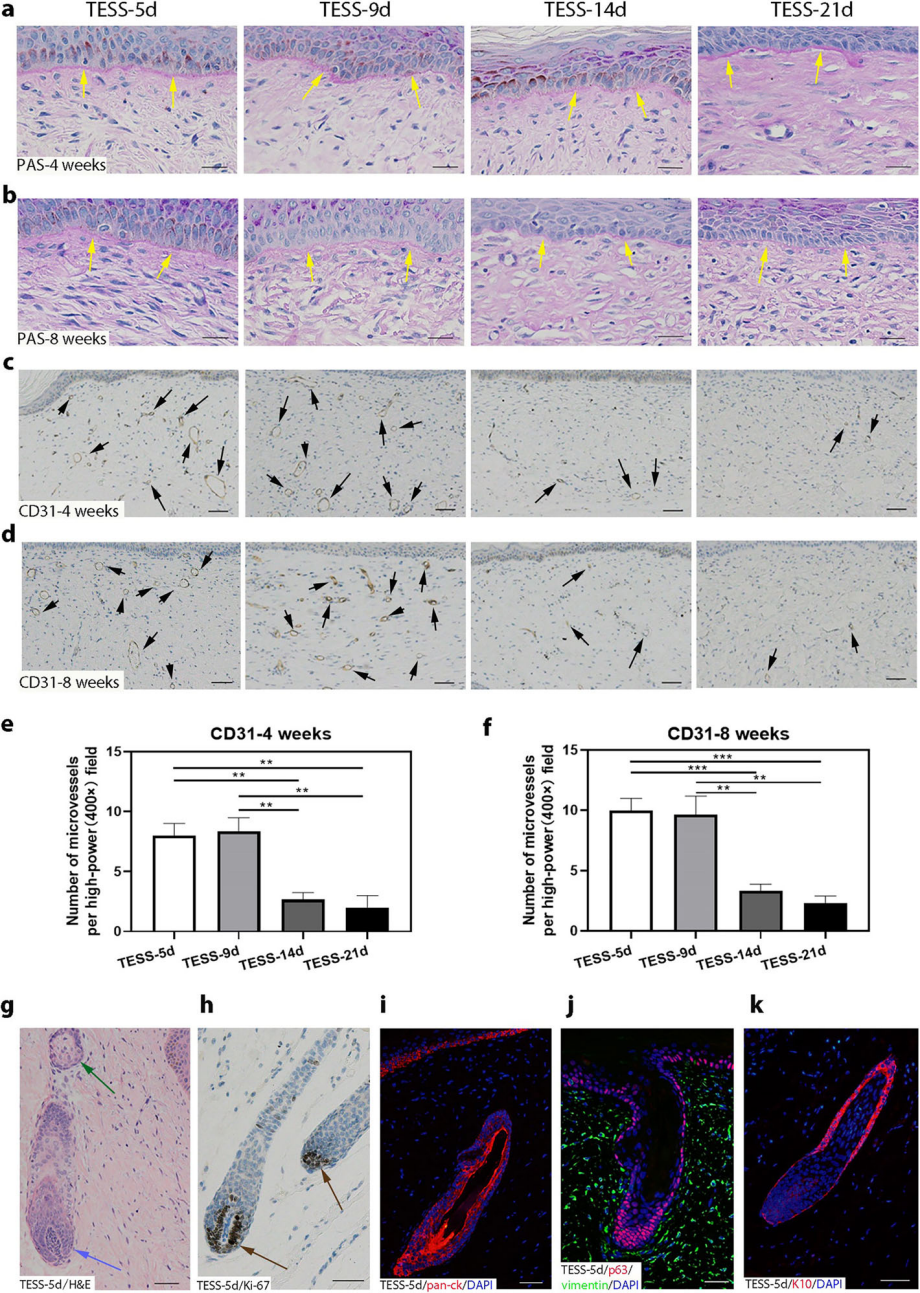
More CD31-positive cells and hair follicles are formed in the dermis of early-stage TESSs. **a**, **b** PAS staining at 4 (**a**) and 8 (**b**) weeks, the red layer shows the location and clarity of the basement membrane zone (yellow arrows). **c**, **d** CD31-positive microvessels (black arrows) were widely distributed in newly formed tissues from early-stage TESSs, while the microvessels were scattered in tissues from TESS-14d and TESS-21d. **e**, **f** Quantification of CD31-positive microvessels per high-power field (× 400) in a light microscope from **c** for 4 and **d** for 8 weeks after transplantation. **p < 0.01, ***p < 0.005 when two groups were compared as indicated; none of the other comparisons was significantly different (3 mice for each group, *n* = 3); bars = 50 μm. **g**–**k** Representative images of hair follicles (H&E staining, Ki-67, pan-ck, p63-vimentin, K10) in the skin formed from transplants TESS-5d at 8 weeks after grafting. The blue arrow in **g** indicates the dermal papilla, and the green arrow in **g** indicates a sebaceous gland. Ki-67 staining demonstrates an active hair matrix with highly proliferating cells (brown arrows in **h**); bars = 50 μm

The integrity of the skin structure requires an intact basement membrane which adheres the epidermis and the dermis together. PAS staining was carried out to identify the basement membrane in the skin grafts formed (Fig. 5a, b) and revealed a continuous line (basement membrane, red layer, indicated by yellow arrows) that was formed at the border of the epidermis and the dermis of all grafts. A stronger and smoother staining pattern of PAS appeared in skin grafts from early-stage TESSs (yellow arrows) but was not significantly different from late-stage TESSs. It is known that vascularization plays an essential role in the survival of grafts after transplantation. IHC staining of CD31, an endothelial cell marker, was performed to evaluate the vascularization in the dermis formed. We found that CD31-positive cells were evenly distributed in the dermis of all 4 groups, and more microvessels (black arrows) were formed in early-stage TESSs after grafting than in late-stage TESSs (Fig. 5c, d), which was verified by quantification of CD31-positive microvessels formed in the dermis, and there was no significant difference between TESS-5d and TESS-9d (Fig. 5e, f).

### Hair follicle formation is only observed in the skin formed from early-stage TESSs

Currently, one of the main problems for treatment with TESSs is the lack of skin appendages such as hair follicles. Interestingly, some hair follicle formation was observed in grafts from early-stage TESSs at 8 weeks after transplantation by histological analysis (Fig. 5g). The hair follicles contained a dermal papilla (blue arrow) and were associated with sebaceous glands (green arrow), and Ki-67 staining demonstrated an active hair matrix with high numbers of proliferating cells (brown arrows, Fig. 5h). Staining with the antibody to human pan-ck confirmed that the hair follicle epidermal cells formed were derived from human cells (Fig. 5i), and the human origin of mesenchymal cells surrounding the hair follicles, stained with p63, was also verified (Fig. 5j). Staining of K10 was localized in the epithelial sheath as a typical epidermal differentiation marker (Fig. 5k). By analysis of all 6 grafts to quantify hair follicle formation, we found that 4 out of a total of 6 grafts (around 65%) formed hair follicles in the transplants of TESS-5d, and 1 of 6 grafts (16%) in the transplants of TESS-9d, but no hair follicle formation was found in the transplants of TESS-14d or TESS-21d. Taken together, these results suggested that cells in early-stage TESSs, especially TESS-5d, have a greater potential to develop into hair follicles compared with late-stage TESSs.

### Early-stage TESSs express high levels of p63 and have less activation of the apoptotic pathway before transplantation

**Fig. 6:**
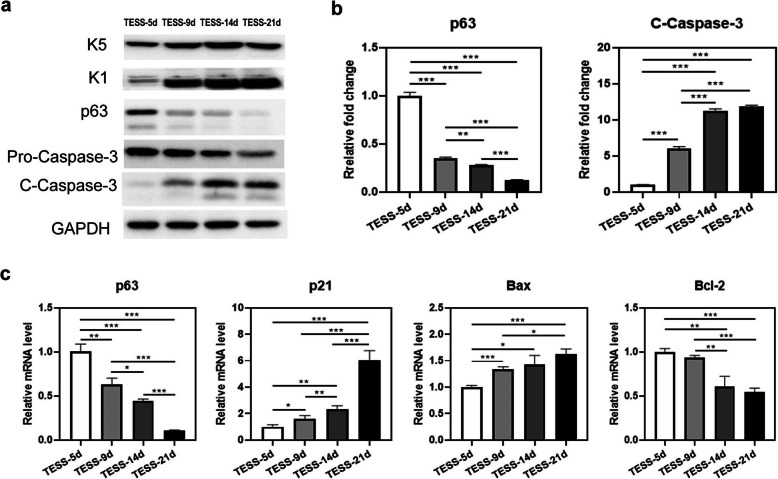
Early-stage TESS expresses high levels of p63, but less activation of the apoptotic pathway. **a** Different stages of TESSs were collected before in vivo grafting for western blot analysis of protein levels of K5, K1, p63, Pro-Caspase-3, and C-Caspase-3. The housekeeping gene GAPDH was used as a loading control. **b** Quantification of western blot analysis of p63 and C-Caspase-3 relative to the band density of GAPDH. **c** TESSs as described for **a** were collected for qRT-PCR analysis of p63, p21, Bax, and Bcl-2. The relative mRNA level of each gene was calculated relative to TESS-5d (expression level as 1) after adjustment by the housekeeping gene 36B4. All experiments were repeated 3 times. *p < 0.05, **p < 0.01, ***p < 0.005 when two groups were compared; none of the other comparisons was significantly different

In order to understand the potential underlying mechanism of early-stage TESSs to produce a better structure of skin after transplantation, TESSs at 5, 9, 14, and 21 days were collected before grafting for western blot analysis of their expression of various differentiation markers, p63, and markers of cell apoptosis (Fig. 6a). The expression of the basal cell marker keratin 5 (K5) was not different among the 4 groups, but the expression of the suprabasal marker keratin 1 (K1) was significantly enhanced with increased culture time in vitro. Importantly, TESS-5d expressed a much higher level of p63, which is crucial for the initiation of epithelial stratification and the maintenance of basal keratinocyte proliferation, compared with the other groups. The expression of p63 was downregulated with increased culture time in vitro (Fig. 6a, b), which was consistent with the in vivo results of p63 staining (Fig. 3c, g). Finally, the expression of cleaved Caspase-3 (C-Caspase-3) was increased with a longer culture time, indicating the activation of cellular apoptosis during the in vitro culture (Fig. 6a), while the activated form of Pro-Caspase-3 (Total Caspase-3) was significantly increased, and the level of Pro-Caspase-3 was reduced correspondingly (Fig. 6a, b). This was further confirmed by qRT-PCR analysis of the expression of the apoptotic markers p21 and Bax, whose expression was increased in late-stage TESSs, and the expression of Bcl-2, an anti-apoptotic gene, was decreased in late-stage TESSs (Fig. 6c). These results suggested that early-stage TESSs contain more undifferentiated cells with higher expression levels of p63 and fewer apoptotic cells compared to late-stage TESSs.

## Discussion

Our study demonstrates that early-stage bilayer TESSs with short-term culture produce a better structure of skin after transplantation in vivo, which potentially benefits better healing for the treatment of wounds. Skin tissue engineering studies started 3 decades ago [36, 37], and autologous epidermal sheets were the first TESSs to be used clinically for the successful treatment of skin burns and played life-saving roles in the 1980s [5]. More recently, using that approach, autologous genetically modified TESSs after transplantation successfully replaced mutant cells to cure epidermolysis bullosa, a genetic skin disorder characterized by the easy formation of blisters [38]. Therefore, TESSs have provided a prospective source of advanced therapies not only for the treatment of acute and chronic skin wounds, but also for genetic skin disorders [7, 8]. So far, based on different demands, different kinds of TESSs have been developed that can be categorized into three groups: epithelial, dermal, and epidermal/dermal (bilayer) replacement materials associated with either analogous or autologous cells [9]. Autologous bilayers (epidermal/dermal layers) of TESSs have been highly recommended for clinical applications because they not only contain full-thickness skin but also can form permanent engraftments after transplantation without immune rejection [7, 39, 40]. However, there are several limitations, including the lack of skin appendages and the time requirements, for autologous bilayer TESSs to be widely used in the clinic [6, 11, 14, 15].

The application of stem cells, which are cells with high potential to proliferate and differentiate, has been proven to play essential roles in the regeneration of skin structures both in in vitro skin models and in in vivo wound healing [38, 41], and more recently, an entire hair-bearing human skin was generated in organoids using embryonic stem cells [42]. Therefore, skin progenitor cells, including scalp-derived dermal progenitor cells and epidermal stem cells, which were prepared in our laboratory and have already been shown to possess the capacity to generate full-thickness skin containing skin appendages, including hair follicles and sebaceous glands [15, 22, 29, 31], were used to reconstitute bilayer TESSs in the present study. TESSs collected at early-stage (day 5 and day 9) and at late-stage (day 14 and day 21) were grafted on full-thickness wound areas of mouse dorsal skin to cover the wounds. Four weeks after grafting, all reconstituted skins formed from transplants of different stages of TESSs had smoothly integrated with the host mouse skin (Fig. 1b–e), and completely covered the wound area (Fig. 1b). All skins formed contained a stratified pigmented epidermis with a normal differentiated structure, which is important for the barrier function, a normal extracellular matrix (ECM) deposited on the dermis and an intact basement membrane between them, which is essential for the integrity of the skin formed (Figs. 1, 2, 4, and 5). These results demonstrate that early-stage TESSs, with as little as 5 days of culture (TESS-5d), are able to produce a comparable structure of the skin as is formed by late-stage mature TESSs (TESS-14d and TESS-21d) at 4 weeks after transplantation.

Surprisingly, further analysis revealed that several features of the skin formed from early-stage TESSs were better than those formed from late-stage TESSs: (1) The size of grafts taken in the group of transplanted early-stage TESSs was larger than those of late-stage TESSs (Fig. 1b, f, g), which suggests that the shrinkage or contracture of early-stage TESSs after grafting was likely less than that of late-stage TESSs. The contracture of skin grafts is a crucial factor that affects the efficacy of wound healing; therefore, it is important to minimize the contracture or shrinkage of grafts which will benefit wound care [43, 44]. (2) The thickness of the dermis formed from early-stage TESSs was greater than that formed from late-stage TESSs (Fig. 4a–d). The density of dermal fibroblasts, the main cell component of the dermis recognized by the vimentin antibody, was higher in the dermis formed from early-stage TESSs (Fig. 4e–h). There were no significant differences in the density of collagen fibers, the main structural element of the ECM, produced by dermal fibroblasts, among all groups, although we observed slightly denser collagen fibers in the dermis formed from the late-stage TESS-21d (Fig. 4i, j). This could be due to some collagen fibers already produced in the TESS-21d during in vitro culture before grafting, since previous studies showed significant collagen deposition in cultures of late-stage TESSs [45, 46]. Concerning the important role of the dermis in skin regeneration, the reconstitution of a better structure of the dermis will significantly improve the quantity of skin wound healing and will reduce scar formation [47]. (3) The number of microvessels in the dermis formed from early-stage TESSs was more than those formed from late-stage TESSs (Fig. 5c–f). Insufficient vascularization is a major threat to the clinical use of TESSs, as it can cause the TESSs to loosen, become susceptible to infection, or experience partial necrosis [48]. The greater vascularization in early-stage TESSs definitely could enhance the efficiency of grafts taken (Fig. 1b, f, g). (4) Notably, the mature hair formation associated with the sebaceous glands were only observed in grafts from early-stage TESSs (Fig. 5g–k). The formation of skin appendages such as hair follicles and sebaceous glands from grafts of TESSs provides a potential opportunity to reconstitute a fully functional skin, which is the ultimate goal for repairing wounds using TESSs. Based on these findings, we conclude that early-stage TESSs produce a better skin structure after transplantation in vivo compared to late-stage TESSs. In addition, the overall structure of the skin formed from TESS-5d was comparable to that formed from TESS-9d; however, the efficiency of hair follicle formation was significantly higher in the TESS-5d, suggesting that the TESS-5d is better than TESS-9d to potentially produce a functional skin.

Our findings indicate that early-stage TESSs likely consist of high potential cells, which possess a high capacity to proliferate and differentiate after transplantation in vivo. Indeed, Ki-67 staining revealed that there were more proliferating epidermal cells in grafts of early-stage TESSs (Fig. 3a, b, e, f), which also could explain how early-stage TESSs with a monolayer of the epidermis at 4 weeks after transplantation could produce a similar thickness of the epidermis as grafts of late-stage TESSs. The strong staining of the epidermal progenitor cell marker p63 (Fig. 3c, d, g, h), which is essential for the proliferation, stratification, and differentiation of skin epidermal cells [49, 50], and the higher expression level of p63 in early-stage TESSs was also verified by in vitro analysis (Fig. 6), which suggested that cells in early-stage TESSs have more potential than cells in late-stage TESSs. A previous study by Larouche et al. reported that hair formation was observed when grafting TESSs formed from newborn mouse hair buds together with mouse dermal cells; however, the number of normal hair follicles was significantly decreased with longer culture time [51]. Taken together, these results suggest that with a longer culture time, there are fewer trichogenic cells in TESSs that fail to reconstitute skin appendages. Moreover, in vitro analysis of the expression of genes involved in apoptosis pathways, including the anti-apoptotic protein Bcl-2 [52] and the proapoptotic proteins p21 and Bax [53–55], in different stages of TESSs, revealed a higher expression of Bcl-2, but a lower expression of p21 and Bax in early-stage TESSs compared with late-stage TESSs (Fig. 6). These results indicate an increased activation of apoptosis with increased culture time, which was confirmed by analysis of cleaved Caspase-3, a key factor in apoptosis execution [56]. These data again support the benefit of transplanting early-stage TESSs for the treatment of wounds.

Currently, late-stage mature bilayer TESSs, which usually require at least 1 week of culture after lifting to the air-liquid interface to mature (see Additional file: Figure S1), have been mainly used for grafting in vivo (both for animal models and for clinical use) [57]. To our knowledge, little research has been done to study whether early-stage TESSs with minimal development of the epidermal layer, which require much less culture time, could form a normal skin structure in vivo after transplantation. Shortening the preparation time of TESSs will not only meet the requirements of clinical application in a timely manner, but will also reduce the cost of producing TESSs. The present study is the first to demonstrate that early-stage TESSs can form hair-bearing skin after transplantation in vivo, which provides an opportunity to deliver autologous TESSs for clinical application up to 2 weeks earlier than the current procedure for the treatment of wounds.

The ultimate goal of tissue engineering skin is to rapidly produce a construct that offers the complete regeneration of functional skin, including all layers (epidermis, dermis, and subcutaneous tissue), all skin appendages (hair follicles, sweat glands, and sensory organs), and a functional vascular and nerve network [6, 58]. Our system still has a lot of room to develop further to reach the above goals since we have not observed any formation of sweat glands or a fat layer in our grafts so far. Therefore, our future work will focus on optimizing our system by applying modern medical technologies such as three dimensional (3D) bioprinting and/or adding different stem cell sources such as adipose-derived mesenchymal stem cells or growth factors, which would promote the regeneration of skin appendages, to quickly and efficiently reconstitute advanced TESSs with the potential to form fully functional skin in vivo after transplantation.

## Conclusions

In summary, the present study demonstrates that early-stage bilayer TESSs, reconstituted from adult skin progenitor cells, contain more competent cells with less activation of the apoptotic pathway and produce a better structure of the skin including hair follicle formation after transplantation compared to late-stage TESSs. These results suggest that the development of early-stage TESSs, which require much less culture time, potentially provides a better healing for the treatment of wounds and promotes its application in the clinic in the future.

## Supplementary information

**Additional file 1: Figure S1.** Schematic of TESS construction, collection, analysis and evaluation. **Figure S2.** Procedure for grafting TESSs in vivo. **Table S1.** Detailed culture information for each stage of TESS. **Table S2.** Oligo sequences used for qRT-PCR analysis.

## Data Availability

The dataset used and/or analyzed during the current study are available from the corresponding author upon reasonable request.

## Abbreviations

3D: Three dimensional
BCA: Bicinchoninic acid
C-Caspase-3: Cleaved Caspase-3
ECM: Extracellular matrix
FBS: Fetal bovine serum
H&E: Hematoxylin and eosin
IF: Immunofluorescence
IHC: Immunohistochemistry
iPSCs: Induced pluripotent stem cells
K1: Keratin 1
K10: Keratin 10
K5: Keratin 5
pan-ck: Pan-cytokeratin
PAS: Periodic acid Schiff
PBS: Phosphate-buffered saline
PFA: Paraformaldehyde
PMSF: Phenylmethylsulfonyl fluoride
PVDF: Polyvinylidene fluoride
RIPA: Radio immunoprecipitation assay
SEMs: Standard errors of the means
TESS: Tissue-engineered skin substitute
