# Supplementary material for: Early-stage bilayer tissue-engineered skin substitute formed by adult skin progenitor cells produces an improved skin structure in vivo
Source: Stem Cell Res Ther. 2020 Sep 18;11:407. doi: 10.1186/s13287-020-01924-z (PMC7501683; doi:10.1186/s13287-020-01924-z)
Supplement: Supplementary file 1 — Additional file 1: Figure S1. Schematic of TESS construction, collection, analysis and evaluation. Figure S2. Procedure for grafting TESSs in vivo. Table S1. Detailed culture information for each stage of TESS. Table S2. Oligo sequences used for qRT-PCR analysis. [file 13287_2020_1924_MOESM1_ESM.docx]

**Supplemental Figures, Figure Legends and Supplemental Tables**

**
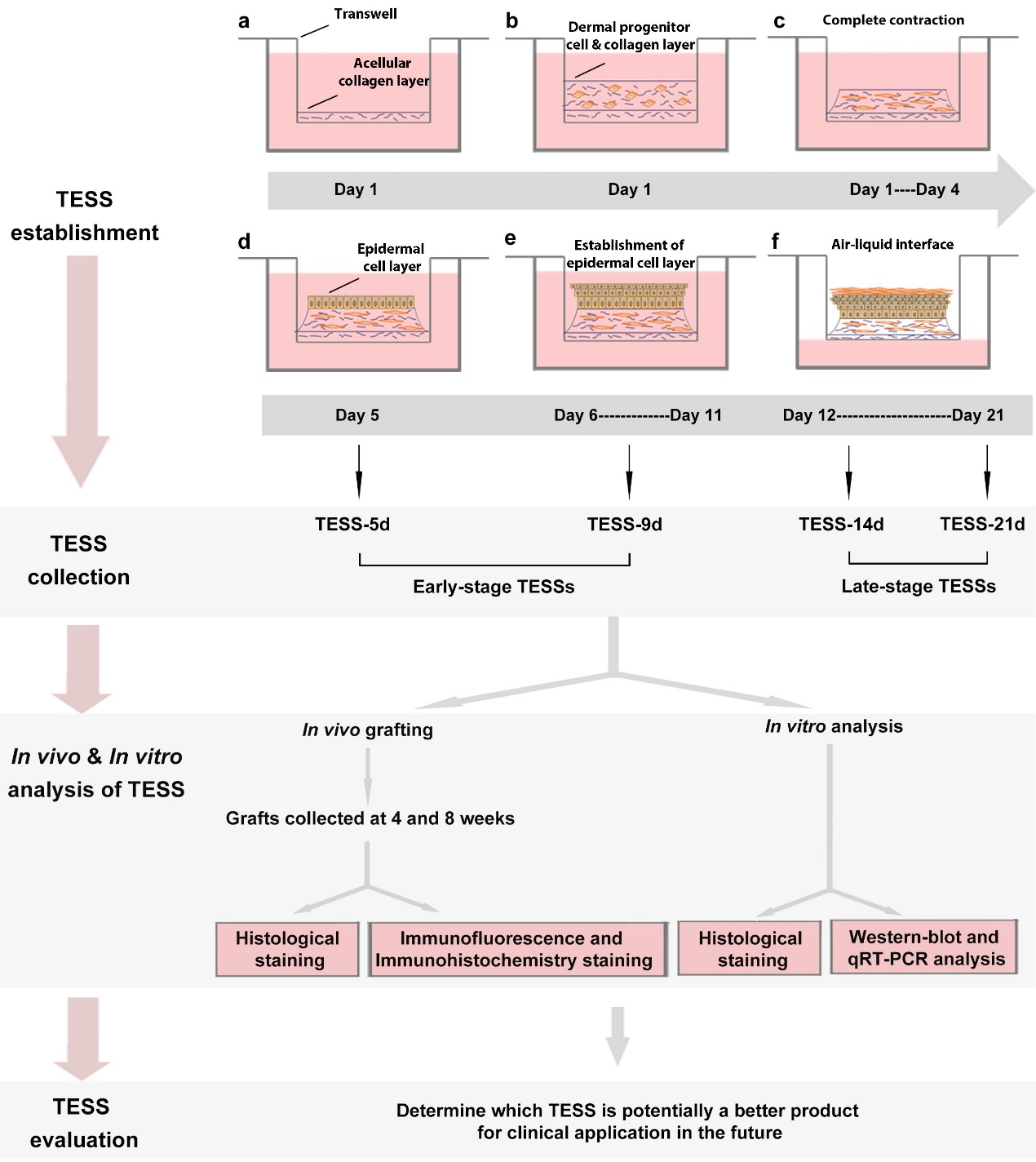
**

**Figure S1.** **Schematic of TESS construction, collection, analysis and evaluation.** TESSs establishment in **a-f**: On Day 1, a thin acellular layer of collagen was constructed. **b** On Day 1, a collagen gel embedded with scalp dermal progenitor cells was layered onto the acellular layer. **c** On Days 1-4, the two layers were submerged in medium for 4 days, and the contracted collagen forms a plateau. **d** On Day 5, epidermal stem cells were added to the center of the plateau of the contracted collagen and allowed to attach to the collagen to create a monolayer. **e** On Days 6-11, the tissues were submerged in conditioned medium, epidermal cells stratify and differentiate and form a suprabasal layer that mimics *in vivo* skin both morphologically and biochemically. **f** On Days 12-21, the tissues were switched to the air-liquid interface to initiate stratification. Further exposure to the cornification medium results in an increase in the thickness of the spinous and cornified layers for maturation of TESSs until 21 days. TESSs at different time points during the culture as shown were collected for *in vivo* grafting and *in vitro* analysis. The grafts were collected at 4 and 8 weeks and were processed for histological and immunochemistry staining. Finally, the TESSs were evaluated to determine which group of TESSs was more suitable for treatment of skin wounds in the clinic.


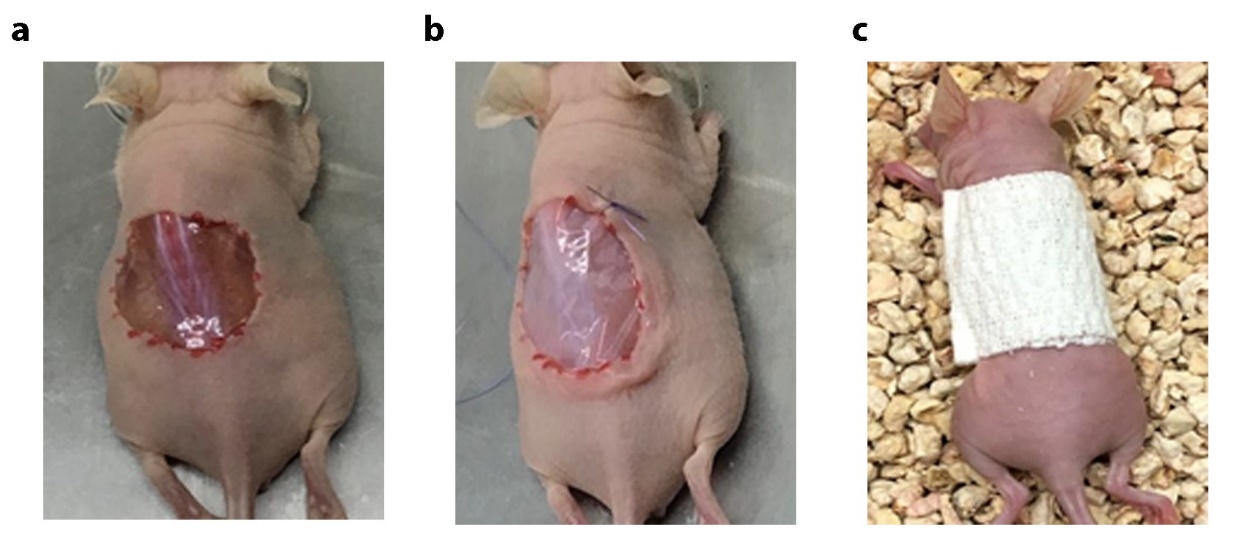


**Figure S2.** **Procedure for grafting TESSs *in vivo*. a** Full-thickness skin was excised according to the size of the TESS on the dorsal skin of nude/nude mice. **b** The full-thickness wound was covered by the TESS attached to a silicon membrane and the graft was sutured into the host mouse skin. **c** The graft was wrapped with a bandage containing vaseline.

**Table S1. Detailed culture information for each stage of TESS**

| Group  Name | | Dermis-collagen Layer Culture | | Epidermal Cell Culture | | Epidermalization  Medium  Culture | | Air-liquid Interface  Culture | | Total  Time |  |
| --- | --- | --- | --- | --- | --- | --- | --- | --- | --- | --- | --- |
| TESS-5 d | 4 days | | 1 day | |  | |  | | 5 days | | |
| TESS-9 d | 4 days | | 1 day | | 4 days | |  | | 9 days | | |
| TESS-14 d | 4 days | | 1 day | | 6 days | | 3 days | | 14 days | | |
| TESS-21 d | 4 days | | 1 day | | 6 days | | 10 days | | 21 days | | |

**Table S2. Oligo sequences used for qRT-PCR analysis**

| **Gene Name** | **Forward** | **Reverse** |
| --- | --- | --- |
| **p63** | GGTCGAAGCGGAGTACTGTC | GCATCGTTTCACAACCTCG |
| **p21** | TGCCCAAGCTCTACCTTCC | CAGGTCCACATGGTCTTCCT |
| **Bcl-2** | TTCTTTGAGTTCGGTGGGGTC | TGCATATTTGTTTGGGGCAGG |
| **Bax** | TCCACCAAGAAGCTGAGCGAG | GTCCAGCCCATGATGGTTCT |
| **36B4** | GCAATGTTGCCAGTGTCTGT | GCCTTGACCTTTTCAGCAAG |
